# Geopolymer Ceramic Application: A Review on Mix Design, Properties and Reinforcement Enhancement

**DOI:** 10.3390/ma15217567

**Published:** 2022-10-28

**Authors:** Nurul Aida Mohd Mortar, Mohd Mustafa Al Bakri Abdullah, Rafiza Abdul Razak, Shayfull Zamree Abd Rahim, Ikmal Hakem Aziz, Marcin Nabiałek, Ramadhansyah Putra Jaya, Augustin Semenescu, Rosnita Mohamed, Mohd Fathullah Ghazali

**Affiliations:** 1 Faculty of Chemical Engineering and Technology, Universiti Malaysia Perlis, Arau 02600, Malaysia; 2 Center of Excellence Geopolymer and Green Technology (CEGeoGTech), Universiti Malaysia Perlis, Kangar 01000, Malaysia; 3 Faculty of Civil Engineering and Technology, Universiti Malaysia Perlis, Kangar 01000, Malaysia; 4 Faculty of Mechanical Engineering and Technology, Universiti Malaysia Perlis, Kangar 01000, Malaysia; 5 Department of Physics, Częstochowa University of Technology, 42-201 Częstochowa, Poland; 6 Faculty of Civil Engineering Technology, Universiti Malaysia Pahang, Kuantan 26300, Malaysia; 7 Faculty of Materials Science and Engineering, University POLITEHNICA Bucharest, 313 Splaiul Independentei, 060042 Bucharest, Romania

**Keywords:** geopolymer, kaolin, ceramic, zirconia, reinforcement

## Abstract

Geopolymers have been intensively explored over the past several decades and considered as green materials and may be synthesised from natural sources and wastes. Global attention has been generated by the use of kaolin and calcined kaolin in the production of ceramics, green cement, and concrete for the construction industry and composite materials. The previous findings on ceramic geopolymer mix design and factors affecting their suitability as green ceramics are reviewed. It has been found that kaolin offers significant benefit for ceramic geopolymer applications, including excellent chemical resistance, good mechanical properties, and good thermal properties that allow it to sinter at a low temperature, 200 °C. The review showed that ceramic geopolymers can be made from kaolin with a low calcination temperature that have similar properties to those made from high calcined temperature. However, the choice of alkali activator and chemical composition should be carefully investigated, especially under normal curing conditions, 27 °C. A comprehensive review of the properties of kaolin ceramic geopolymers is also presented, including compressive strength, chemical composition, morphological, and phase analysis. This review also highlights recent findings on the range of sintering temperature in the ceramic geopolymer field which should be performed between 600 °C and 1200 °C. A brief understanding of kaolin geopolymers with a few types of reinforcement towards property enhancement were covered. To improve toughness, the role of zirconia was highlighted. The addition of zirconia between 10% and 40% in geopolymer materials promises better properties and the mechanism reaction is presented. Findings from the review should be used to identify potential strategies that could develop the performance of the kaolin ceramic geopolymers industry in the electronics industry, cement, and biomedical materials.

## 1. Introduction

Geopolymer manufactured raw materials are extremely rich in silica and alumina, which is an advantage given that over 65% of the Earth’s crust is composed of alumina and silica minerals [1,2]. Geopolymer consists of a three-dimensional network of aluminosilicate tetrahedral atoms that are covalently bound to one another [3,4,5,6]. Geopolymers are a relatively recent type of construction material created from industrial by-products and cementitious materials with high alumina and silica content [1,6,7,8]. Due to climate change strategic initiatives, the market for geopolymer products has expanded dramatically in recent years. In addition, green systems are a key problem in the building industry, and the use of geopolymers through the geopolymerisation process has piqued the interest of scientists worldwide [5,6,9,10]. It is a “new” category of materials that has attracted a great deal of interest and risen gradually in research article investigations over the past decade.

Geopolymers have traditionally been considered an alternative to Portland cement-based materials with significant environmental and durability benefits. These advantages need to be compensated for by many brief mix designs and technologies when compared to conventional Portland cement [7,11,12]. Blended cements use a wide variety of non-conventional ingredients, such as geopolymer binders and pozzolan-based compounds. However, geopolymers have many additional potential uses; for example, that are advantageous due to thermal stability in the fabrication of thermally resistant structural elements [5,13,14,15], as adhesives [16,17], for the solidification of hazardous wastes [18,19,20,21], or as catalytic support [7,8,22]. 

Blended cements are stronger and less likely to crack than conventional cement, not to mention being eco-friendlier. Inorganic geopolymers are synthesised in an alkaline environment from silica–alumina gels [6,7,9,13,23]. When viewed with scanning electron microscopy (SEM), the structure is composed of interconnected chains or networks of inorganic molecules that are held together by covalent bonds. One atom of silicon or aluminium is connected to four atoms of oxygen to form a tetrahedron. These tetrahedrons form a three-dimensional network with one oxygen atom in common between each of the tetrahedrons [17,18,23,24,25]. The most used raw materials are natural minerals, such as kaolin [9,24,26,27] and calcined clays [28,29,30,31,32], and industrial wastes, such as fly ash [4,13,33,34,35,36], slag [35,36,37], red mud [28,38,39], and waste glass [40,41,42]. 

Kaolin converts to a pozzolan material named metakaolin (MK) after high temperature of thermal treatment. Regarding the issues of sustainability, kaolin as a geopolymer material can satisfy the world demand for ceramic industries. This review also discovered findings on the potential use of kaolin as a raw material with and without thermal treatment. However, there have only been a few research studies conducted on the use of kaolin as a raw material in a ceramic geopolymer application. This article also discussed a comprehensive review of the characterization of kaolin, addition of kaolin geopolymer, and the potential of zirconia reinforcement in ceramics. Furthermore, the experimental results by the researchers regarding the percentage ratio of zirconia addition to improve the properties of the ceramic geopolymers are also presented. At the conclusion of this review, the feasibility of future research into the low-cost manufacture of ceramics from geopolymer derived from kaolin is evaluated. Therefore, it is necessary to undertake a thorough literature analysis on current understandings regarding the functionality of geopolymer regarding its application on ceramic.

## 2. Mix Design and Manufacture Method for Kaolin Geopolymer in Ceramics

A new type of building materials with improved strength, durability, and other qualities entered the market in the nineteenth and twentieth centuries [43,44]. Ceramic components can be made from a wide variety of metallic and non-metallic atom combinations, and each atom combination typically lends itself to a number of structural configurations [30,40,45,46]. To address the rising needs and requirements in a wide range of application fields, scientists were compelled to develop numerous novel ceramic materials.

Inorganic solid powders with carefully controlled purity, particle size, and particle dispersion are used to create ceramic geopolymers [9,17,22]. To create a ceramic with specific material properties, various precursors are mixed in the process. This powdered mixture is mixed with a binder so that it can be machined in a “raw” state, moulded to exact specifications, and then sintered in a controlled furnace [40,41,47]. The raw ceramic must be heated to a temperature below its melting point to be sintered. By removing the moisture and binder, fine ceramic products with high hardness and density are created by condensing the microscopic gaps between the particles and fusing them together [30,46,48]. The formulation of geopolymer materials for ceramic applications is shown in Table 1.

**Figure 1:**
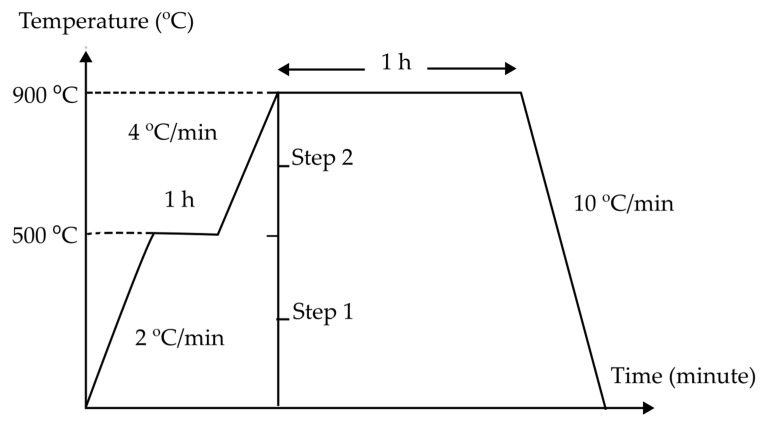
Two-steps sintering profile of kaolin-GGBS geopolymer [22].

According to Jamil et al. [22], the phase transition of the sintered kaolin-ground granulated blast surface slag (GGBS) geopolymer was aided by the addition of GGBS to kaolin, which accelerated the geopolymer’s setting time. Kaolin’s structural alterations were influenced by the high alkalinity of NaOH (8 M), which made it capable of reacting with GGBS. The sintered kaolin-slag geopolymer’s characteristics alter as the solid to liquid (SL) ratio rises. Akermanite and albite are two new phases that are formed when the solid content is at its highest (SL:2). The morphology of the sintered kaolin-GGBS geopolymer indicates enhanced densification and pore creation with increasing solid-to-liquid ratios. Additionally, two steps of the sintering profile, as shown in Figure 1, mitigated the beginning of fractures as the dihydroxylation mechanism is retarded. In this research, the use of kaolin as a raw material without calcination gives good feedback on energy consumption and green method by skipping the sintering stage. The effect of kaolin geopolymer at post-sintering temperatures, however, is not explored further in the thermal gravimetric and thermal analysis.

**Figure 2:**
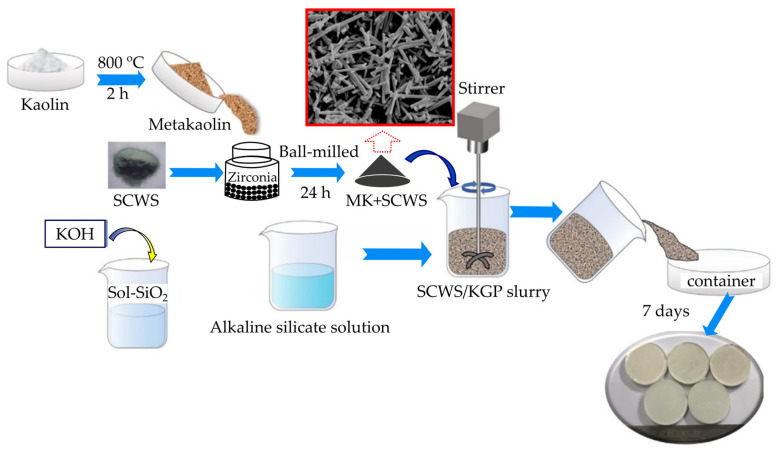
Preparation procedure for SCWS (SiC whiskers)/KGP (Kaolin Geopolymer) composite [46].

Ma et al. [46] revealed that the flexural strength SiC whiskers (SCWS) reinforced geopolymer composites (SCWS/KGP) composites could be improved with the presence of SiC whisker and reached the peak value when the SCWS content was 2 wt%. The production process for the composite of SiC whiskers (SCWS) and KGP (Kaolin Geopolymer) is shown in Figure 2. The improvement in the KGP composites’ flexural strength is mostly attributable to the strong interface bonding between the SiC whiskers and the geopolymer matrix. When its content reached 4 wt%, whiskers aggregation was observed, which negatively impacted the mechanical performance of SCWS/KGP composites. Additionally, geopolymer evolved into high density, twin-structure leucite ceramics after being heated to 1100 °C and 1200 °C. While this was going on, there was no interfacial reaction between the leucite matrix and the SiC whisker, which preserved its chemical stability. Due to leucite formation and a strong interfacial contact between the whisker and matrix, the composite treated at 1200 °C with 2 wt% SiC whisker demonstrated a 124.8% higher flexural strength than the composites before high temperature treatment. Nevertheless, this research does not compute the compressive strength, which is the interfacial zone between the whisker and the matrix, because shrinkage can be determined by the whisker that is subjected to compressive stresses.

**Figure 3:**
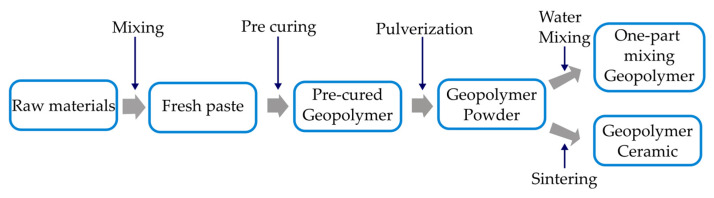
Steps to produce geopolymer powder, one-part-mixing geopolymer and ceramic geopolymer [48].

Yun Ming et al. [48] confirmed the existence of zeolite Y in metakaolin-based geopolymer powder-based geopolymers with one-part mixing. Figure 3 depicts the production procedures for geopolymer powder, one-component geopolymer, and ceramic geopolymers. The one-part mixing geopolymers attained a maximum compressive strength of 10 MPa after 28 days. The sintering of the compressed geopolymer powder changed the amorphous phases into nepheline phases without passing through intermediate phases. At 1200 °C, the greatest flexural strength of ceramic geopolymers was 90 MPa. This method reduced the probability of cracking in geopolymers that had already been cured. However, it was recommended to reduce the sintering temperature to produce nepheline ceramic geopolymers, as the sintering temperature indicated in this study was too high.

## 3. Factors Affecting the Suitability of Kaolin Geopolymers in Ceramics

Alumina is now used in the production of ceramic membranes. Kaolin, which is extensively used as a substitute for alumina due to its unique chemical and physical properties, provides a membrane with low plasticity and high refractoriness [22,30,49]. Additionally, kaolin shows hydrophilic properties. It possesses good chemical and fire resistance in addition to a comparatively high mechanical strength [50,51,52]. Therefore, geopolymers have the potential to be employed as construction and building materials that are environmentally beneficial. Under thermal activation, kaolin geopolymers become more stable, and kaolin clay transforms into the reactive phase of metakaolin. When metakaolin was employed as an aluminosilicate precursor [35,48], its characterization was simplified.

Ceramics cannot transfer high internal loads via plastic deformation due to their brittleness properties [53,54,55]. Despite all its benefits, ceramics as a building material also has several drawbacks. The basic structure of kaolin is a highly disturbed phyllosilicate network consisting primarily silicon and aluminium, which confer the major advantage of having a particle size distribution that is relatively homogeneous [22,50,56,57].

### 3.1. Curing Process

In general, efforts are undertaken to develop ceramics with superior mechanical qualities by incorporating amorphous phases, whiskers, fibres, particles, and even metallic phase and pores. Another method for improving the ballistic impact on a ceramic surface is to promote finer particle size, which prevents the initiation and spread of failures such as pores, flaws, fractures, and cracks [58,59]. Table 2 provides a review of prior research on geopolymer materials for diverse ceramic applications.

Numerous studies of kaolin as a ceramic material show it is widely used for high performance ceramic materials, which are divided according to the end use application into electronic packaging industries [61], electroceramics (dielectric [64], insulator [63]), ceramic foam [62], ceramic grog [9], cementitious materials [15,60], soil construction [65], and ceramic coatings [66].

Kaolin has narrower interlayer spacing and less cation exchange capacity than other clay mineral materials [17,65,67]. Kaolin is the principal clay formed by chemical weathering; it is coarse in particle size and inflexible compared to other clays. It is the most researched clay mineral in this field, and its extensive use is attributed to its capacity to change into the metastable and more reactive phase of metakaolin following dehydroxylation at temperatures between 550 °C and 800 °C [52,62,68]. Similarly, kaolin’s basic structure consists of a highly disturbed phyllosilicate matrix comprising primarily silicon and aluminium, with little variation in particle size [69,70].

### 3.2. Si and Al Composition

Geopolymer materials composed of Si and Al have more potential as ceramics. As one of the most important clay minerals, kaolin has a high porosity, strong mechanical stability, and low thermal conductivity in geopolymers [22,50,71]. During the firing of kaolin, the type and quantity of secondary phases can have a significant impact on the thermal properties of the raw materials [48,68]. Iron oxide is very significant [49,72]; Fe_2_O_3_ can exist in raw materials as either mineral complexes or silicate structures. The addition of Fe_2_O_3_ in kaolin not only increases the quantity of mullite phase at lower temperatures (1050 °C), but also improves the crystallisation of mullite at higher temperatures [19,73,74]. Table 3 lists the Si and Al content of numerous kaolin types successfully employed in geopolymer production.

The percentage content range of Si and Al was from 32.04% to 70.32% and 11.60% to 44.2%, respectively, while the lowest particle size was 1.3 µm and lowest surface area is 1.57 m^2^/g. According to previous research, mechanical activation altered the particle size and specific surface as well as the kaolin’s reactivity with respect to the geopolymerization reaction, hence increasing the compressive strength of the geopolymers [69,81,82,83]. This increase was attributed to the smaller particle size and altered shape, which allowed for a faster dissolution of the particles in the activating solution [82,84]. The initial crystalline structure of clay minerals that already exist is broken during dehydroxylation, making the substance reactive; obviously, the higher the level of dehydroxylation, such as amorphousness, the more reactive the material [22,49,50,76]. Kaolin as a geopolymer material which has high Si Al content is highly suitable in ceramic application

There is a critical alkaline concentration that can achieve the maximum compressive strength, and a higher concentration does not favour the formation of geopolymers, according to prior research that used kaolin as a single source material to synthesise geopolymer products and investigate the effect of different alkaline activator concentrations on the compressive strength [22,56,85,86]. Important criteria that influence the degree of the geopolymerization process and the reaction duration with the alkali activator, respectively, are the Si-to-Al ratio and the ageing time [26,63,87]. Table 4 summarises the utilisation of alkali activator in past studies on kaolin.

The reaction of alkaline activators has been observed; NaOH and Na_2_SiO_3_ are commonly used at a range of 6 to 10 molar NaOH concentration. In the synthesis of kaolin geopolymers, the higher molar concentration of KOH, 16 Molar, is employed. In addition, high concentrations of KOH are utilised to enhance the solubility of Al^3+^ and Si^4+^ ions [26,88].

In addition to the selection of raw materials and manufacturing conditions, geopolymers can exhibit a vast array of qualities and characteristics. In general, the properties of geopolymers are highly reliant on the composition of the reactants, particularly the Si/Al ratio, process, and method, mixing design, and alkali activator type. In addition, the most important component in determining the application sectors of geopolymers is the sintering process, which is the most crucial step in the fabrication of ceramic geopolymers.

### 3.3. Sintering Temperature

**Figure 4:**
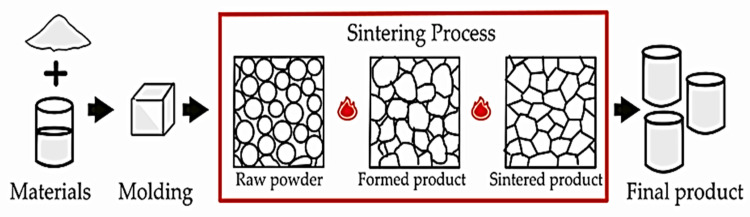
Sintering process flow.

Sintering is the process of producing a solid mass of material under pressure and heat without fully melting it. In this process, atoms in raw materials diffuse across particle boundaries and fuse to form a single solid object. The sintering process flow is shown in Figure 4. 

Zhang et al. [57] revealed, possibly for the first time in China, the alkali activation reactivity of calcined kaolin from Guangxi province. The thermal treatment of kaolin is typically necessary to obtain more reactive precursors that result in geopolymers with high strength was discovered. Significant attempts have been made to identify the best heating temperature for the increase of the kaolin geopolymer’s strength. Past research findings regarding the sintering temperature are presented in Table 5.

Naghsh and Shams [89] demonstrated that as the calcination temperature increased from 400 °C to 800 °C, the MK dissolution extent in NaOH solution increased continuously. Sornlar et al. [90] discovered that dehydroxylation of kaolin to metakaolin occurred upon calcination at 600 °C, resulting in a large increase of amorphous phase with some crystalline phases (illite and quartz) remaining in the resulting metakaolin powder. According to reports, the amount of amorphous phase in the metakaolin had a significant effect on the curing and strength development of the geopolymer. Due to the relatively wide specific surface area, which may necessitate a high water-to-binder ratio to obtain satisfactory workability, MK is not utilised in the majority of construction situations.

Alexandre and Lima [26] conducted additional research using KOH as an alkaline activator. After calcination, minerals such as anatase and quartz in samples sintered at 750 °C remain stable or metastable. In addition, the de-hydroxylation of goethite has led to the neoformation of hematite in these samples. However, current knowledge suggests that the use of KOH as an alkali activator does not optimise geopolymerization, as the K-O link is weaker than the Na-O bond.

Majdoubi et al. [91] showed that the crystalline phase of kaolinite disappears between 300 °C and 1300 °C of sintering temperature. The constant emergence of several characteristic peaks of kaolinite suggests that calcination at temperatures between 700 °C and 800 °C was not performed perfectly. At 800 °C, it is readily apparent that the full absence of the halo characterises the amorphization of our material, indicating that the three-dimensional network of geopolymers is no longer in place and has experienced a significant transformation from amorphous to completely crystalline. When the temperature approaches 1100 °C, the aluminium phosphate phosphocristobalite phase and SiO_2_ cristobalite dominate the X-ray powder diffraction (XRD) graph. This phase is the most resistant at high temperatures, which explains why the geopolymer’s resistance is very weak. The slight variation between the three halos is a result of the increase in calcination temperature: the higher the temperature, the greater the intensity. After heat treatment, it was also noticed that the typical peaks of quartz and muscovite become more distinct and intense.

Merabtene et al. [92] discovered that calcined kaolin at 800 °C for 24 h, followed by quick air cooling and the selection of 800 °C possessed an excellent precursor for geopolymer synthesis. The heating scheme of 800 °C confirms the existence of carbonates such as calcite (CaCO_3_), as indicated by XRD and Fourier transform infrared (FTIR) investigations. Nevertheless, the sintering temperature must be increased to 900 °C due to the increasing kaolin reactivity between 800 °C and 900 °C. It appears to be connected to the altered oxygen atom environment during dehydration.

Villaquirán and Meja [93] determined the sintering temperature to be between 300 °C and 1500 °C. In the absence of dehydration, the compressive strength of the geopolymer reached its maximum value as the calcination temperature rose to 900 °C and decreased drastically at 1000 °C. However, the 300 °C sintering temperature range is not really significant because past research has shown that kaolinite gradually loses OH cation between 700 °C and 900 °C during the sintering temperature.

Jamil et al. [22] emphasised from the outset that 6 M to 8 M of NaOH is sufficient to achieve alkalinity. The partial conversion of Al from its original 4-coordinated state to its 6-coordinated state is known as the phase transformation from monoclinic to tetragonal. As a result, the sintering temperature ranges between 200 °C and 1200 °C. The kaolin geopolymer after pre-sintering, which can be explained by the beginning of crystallisation of the amorphous geopolymer network, the total disappearance of the gehlinite phase, and the beginning of the appearance of the akermanite and albite phase, is due to the phase change from the more stable hexagonal aluminium phosphate. This phase is responsible for the observed colour change; the geopolymer has turned white due to the presence of crystalline Al, which indicates the presence of the akermanite and albite phases. Previous geopolymer research [20] had already revealed this modification.

Liew et al. [48] evaluated the reactivity of kaolin calcined at 900 °C, 1000 °C, 1100 °C and 1200 °C in a furnace at a heating rate of 5 °C/min and soaking duration of 3 h and discovered that the ceramic geopolymers had a maximum flexural strength of 90 MPa at 1200 °C. This study discovered the highest sintering temperature among previous studies. There is, in reality, no universally ideal temperature for MK production. Nevertheless, it is acceptable to select various calcination schemes (temperature and duration) because the mineral composition and particle size of kaolin, as well as the heating procedure, all have a role (stable or fluidized).

The porosity and apparent density variation with kaolin content have different aspects according to the sintering temperature. The sintering reactions between kaolin and alkali activator absolutely influences the chemical and mechanical properties of kaolin ceramic geopolymers, respectively. Although calcination temperature had a positive impact on aluminium alloys, the high calcination temperature had a significant negative impact on the sustainability of the environment. As a comparison, the calcination of kaolin to obtain metakaolin takes place from 600 °C to 800 °C. Concerning the environmental impact and sustainability of geopolymeric cements produced from natural kaolin, the use of non-calcined kaolin aids in the reduction of manufacturing costs and environmental implications, resulting in a green ceramic [2,22,94].

## 4. Properties of the Kaolin Ceramic Geopolymers 

**Figure 5:**
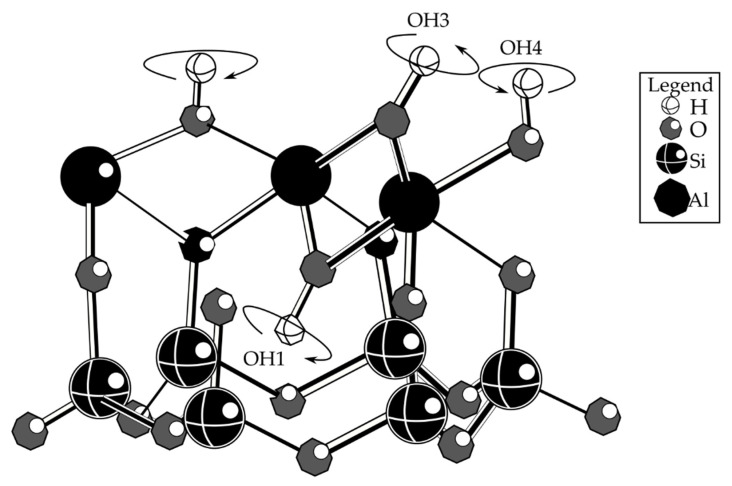
Chemical structure of kaolin [95].

Figure 5 shows that the chemical structure of kaolin in the 3D network structure of geopolymers consists of a Si_2_O_2_Al framework spatially connected chains of [SiO_4_] and [AlO_4_] tetrahedral. The Si and Al share oxygen corners for each other and produce the charge-balancing metal cations.

Kaolin is a common mineral found in soils and sediments, and it has a wide range of applications. This clay mineral is a 1:1 layer aluminosilicate in which an alumina octahedral sheet and a silica tetrahedral sheet are fused to produce a layer held together by hydrogen bonding [73,93,96]. This clay possesses no exchangeable cations [89,97,98], because isomorphic substitution and cationic vacancies are near to zero.

Theoretically, any pozzolanic compound with a high alumina and silica concentration is acceptable for geopolymer synthesis under highly alkaline circumstances [18,49,56,76]. However, several considerations must be made for the geopolymerization reaction following the addition of an alkaline activator. The measurement of the physical properties and chemical composition of the raw material is one of the most essential variables in this category, as it determines the alkalinity level of the activator [19,22]. It is essential to completely analyse the samples and, based on this, to optimise the composition and amount of the activating solution and curing conditions due to the diversity of the raw materials, which may vary from batch to batch, whether mineral or waste products, for example [17,18,99,100].

Clays are hydrous aluminium silicates with a composition of approximately Al_2_O_3_–2SiO_2_–2H_2_O [67,101]. In order to lower costs, contemporary research on the manufacture of ceramic support has centred on the use of less expensive raw materials, such as apatite powder, fly ash, natural raw clay, dolomite, and kaolin. Among these ceramic materials, kaolin has emerged as a potential raw material that is frequently employed for separation applications at a lower cost [57,73]. Moreover, kaolin is one of the least expensive and most abundant support raw materials, and it is readily accessible [22,33,102].

Geopolymers derived from kaolin has demonstrated great promise in the construction and building industries as well as engineering applications. Previous research indicates that changes in the reactivity of source materials employed in the synthesis of waste-based geopolymers have a substantial impact on the final characteristics of the ceramic geopolymer. At the raw materials selection stage, the attributes of kaolin correspond to its mineralogical compositions and thermal treatment histories. Consequently, it merits additional research into its compressive strength, chemical and mineralogical composition, morphological development, and phase analytic features.

### 4.1. Compressive Strength

The compressive strength of a geopolymer is contingent on several factors. These factors include the strength of the gel phase, the ratio of gel phase to undissolved Al-Si particles, the distribution and hardness of the undissolved Al-Si particle size, the amorphous nature of the geopolymer or the degree of crystallinity, and surface reactions between the gel phase and undissolved Al-Si particles [76,103,104,105]. For instance, curing at elevated temperatures for more than two hours appears to promote the development of compressive strength. Nevertheless, curing at 70 °C appears to increase compressive strength significantly more than curing at 30 °C during the same period. Table 6 displays the compressive strength and influencing parameters from prior studies.

**Figure 6:**
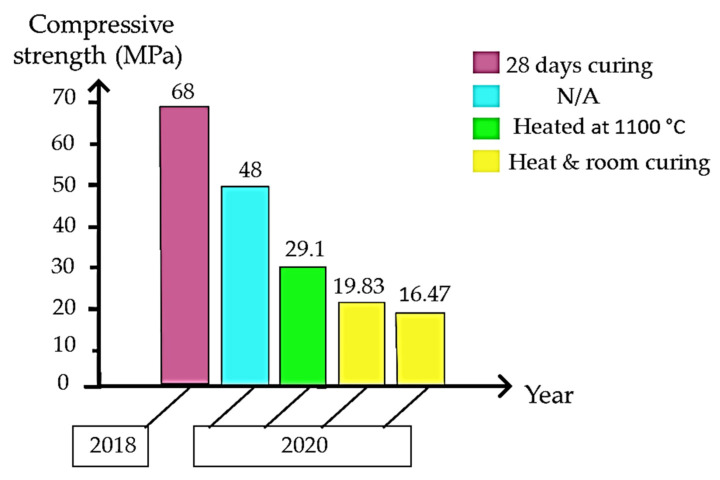
Reported compressive strength using kaolin.

Figure 6 shows the compressive strength using kaolin from prior studies summarized from Table 1. The best compressive strength by using kaolin as raw materials, 68 MPa was obtained at 28 days of curing by using high density water glass about 1.5 g·cm^−3^. Choosing a suitable water glass density and type of alkali activator helps to ensure that the geopolymer mortar used as ceramic has good compressive strength. Generally, the ratio of each binder either solid or liquid will result in different compressive strength due to phase and bond formation in geopolymer system.

### 4.2. Chemical and Mineralogical Composition

The chemical composition of raw materials as determined by X-ray fluorescence impacts the development of geopolymerization and the kind and quantity of zeolite [12,22,63,107]. The primary chemical component of kaolin, kaolinite, is dehydroxylated at temperatures reaching 550 °C, hence converting its long-range organised microstructure to an amorphous state. Consequently, kaolinite has been converted to metakaolin [48,50,74]. In geopolymer synthesis, the kind and temperature of thermal treatment affect the reactivity of metakaolin. Due to its relatively well-defined chemical structure, chemical composition, and properties, which increase its reactivity [50,57], kaolin is also considered one of the most essential precursors for geopolymer synthesis [22,49,50,56,68,108]. According to several studies, the use of kaolin as a raw material for the synthesis of geopolymer is environmentally friendly because it generates less carbon dioxide than the production of Portland cement. Table 7 displays the chemical composition of kaolin geopolymer.

Alumina (Al_2_O_3_) and silica (SiO_2_) content, in general, have the greatest impact on the geopolymerization. Other mineral compositions also play a part, including magnesium oxide, MgO (speeds up the hydration reaction and may cause low porosity and high bulk density due to the large volume of the hydrate) [49], iron(III) oxide, Fe_2_O_3_ (able to exhibit adsorptive, ion-exchange, and catalytic properties similar to those of zeolitic aluminosilicate molecular sieves) [110], and calcium oxide, CaO (acts as to harden at room temperature without affecting the mechanical properties of the final product) [22].

### 4.3. Morphological and Phase Analysis

**Figure 7:**
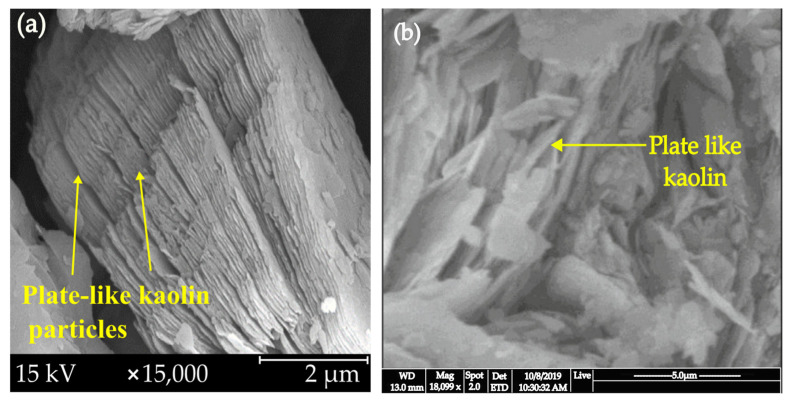
SEM images of the plate like kaolin (**a**) [111] (**b**) [79].

Scanning electron micrographs of kaolin’s microstructure were examined. These show that morphology changes as the calcining temperature rises, and it also gradually affects the strength, hardness, apparent density, and volume shrinkage ratio. Figure 7 shows plate like kaolin SEM micrographs. It is evident that the kaolin morphology consisted of crystals with sharp edges, hexagonal shapes, rods, plates with corrosion, and irregular shapes [22,49,111]. The geopolymer made from kaolin has the benefit of being reliably created, with known properties during both preparation and development. However, the rheological issues caused by its plate-shaped particles make the system more complex to process and require more water [51,111]. 

**Figure 8:**
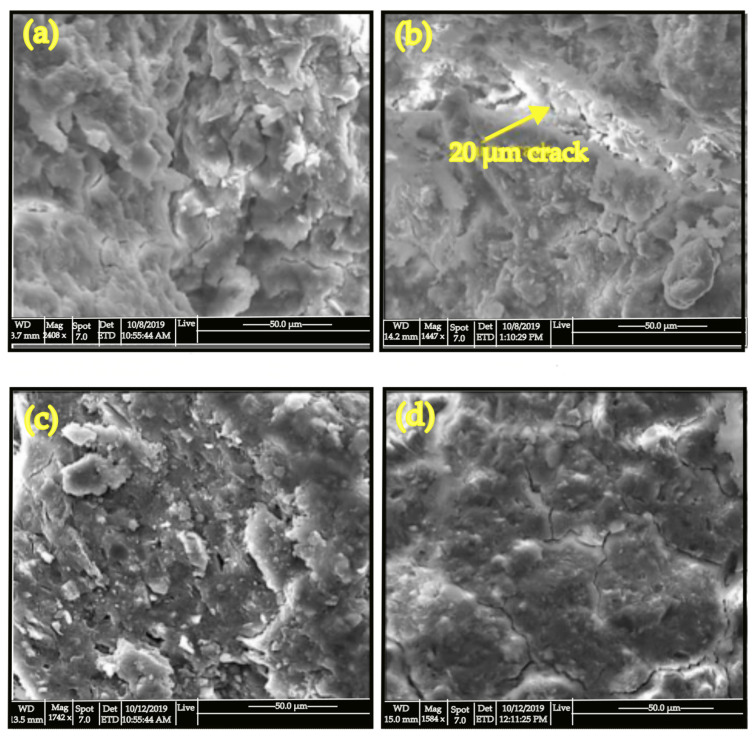
SEM images for (**a**) a room-temperature cured paste, (**b**) paste specimen after immersed in water, (**c**) paste with 10% NaOH and (**d**) paste with 10% CaO [79].

Figure 8 shows the SEM images for the room-temperature cured binders (a), paste specimen after immersed in water (b), room-temperature cured binders with 10% NaOH (c), and for the room temperature cured binders with 10% CaO (d). When the specimen was cured at room temperature, several microcracks with discontinuous gel developed, however a rather big crack was identified in the paste specimen following immersion in water. As illustrated in the figure, this produced a dense and strong alkali aluminosilicate matrix (c). The formation of such cracks could explain why the compressive strength of geopolymer specimens decreased following immersion in water. A solid gel with a well-packed structure was observed in the CaO paste, with visible amounts of crystalline or weakly crystalline C-S-H phase. The inclusion of C-S-H phases may provide stiffness to the geopolymer paste, improving the mechanical properties of a kaolin-based geopolymer [79,99,112]. The addition NaOH to the system could enhance the leaching of Si and Al from the kaolin particles to the solutions and resulted in increased geopolymerization and formation of sodium silicate hydrate gel [23,36,113,114].

The Si/Al ratio has a strong influence on the microstructure of geopolymers, and the other three parameters (Al/Na, water/solids, and H_2_O/Na_2_O ratios) have less of an impact. This is shown by the fact that geopolymers with the same Si/Al ratio have similar microstructures, but there are big differences when the Si/Al ratio changes [25,115,116]. The geopolymer system is a two-phase gel made of water and an aluminosilicate binder. The water acts as a reaction intermediate and is released when the gel solidifies to create pores and a two-phase structure [15,105,117]. In contrast, water plays an active role in cement hydration and ultimately affects paste porosity in the OPC system [49,56,118]. Porosity in geopolymers is determined by solution chemistry during geopolymerization, which is primarily a function of Si/Al ratio and alkali metal cation type. Absolute pore volume is governed by nominal water content [50,64,119].

**Figure 9:**
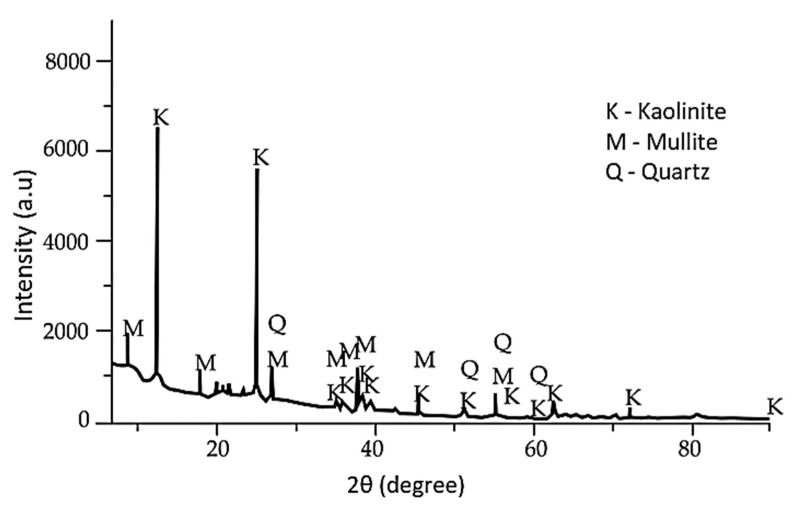
XRD patterns of kaolin (K) [51].

Only three of the kaolin’s classic phases, quartz, muscovite, and kaolinite, can be seen in the XRD diffraction pattern depicted as in Figure 9 [22,51,108]. One of the crystalline phases found in kaolin is kaolinite, which makes up 35% of the crystalline phases and is converted to metakaolin through calcination [35,117,120]. The decomposition of the mineral calcite into CaO and CO_2_, which is evidenced by the mass loss at around 677 °C in the thermogravimetric analysis (TGA), is what causes the calcite reflections in the XRD pattern of the calcined ceramic industrial sludge to vanish [51].

**Figure 10:**
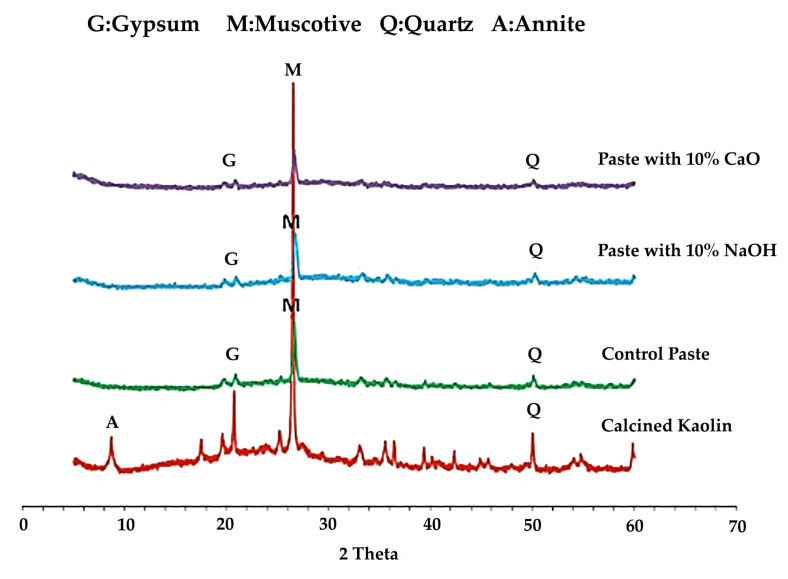
X-ray Diffraction Analysis for kaolin-based geopolymer [79].

The XRD patterns for calcined kaolin and the comparable hydrates with and without additions are shown in Figure 10, where, relative to calcined kaolin, the kaolin-based geopolymer paste has less crystalline peaks. Several crystalline hydrated phases, including quartz (Q), muscovite (M), and gypsum, were discovered (G). The addition of NaOH or CaO has only a minimal impact on the crystalline phases of the resulting hydrates.

Kaolin is highly recommended for use in ceramic geopolymers based on its excellent properties as a ceramic. In addition, the properties of the ceramic geopolymer can be enhanced by addition of reinforcement. There is a critical need for ceramic reinforcement to enhance its physical and mechanical properties. Geopolymers have several positive properties, including high strength, high density, few pores, an elastic modulus, and little shrinkage; yet, brittle and can easily break. Reinforcement or addition in kaolin geopolymers may solve this problem.

## 5. Reinforcement in Kaolin Ceramic Geopolymers

In comparison to OPC-based materials, geopolymers exhibited improved mechanical characteristics and resilience to fire, sulphates, and acids. When used as OPC products, geopolymers, however, exhibit brittle failure due to low tensile strength, which may place several restrictions on its potential structural uses. Usually the material properties of kaolin ceramic geopolymers have a greater impact on the performance of reinforced geopolymer composite than binders do. Table 8 summarises the various types of additions, the percentages of additions, and the results of studies that examined the enhancement of properties in kaolin geopolymer ceramic.

Wu and Tian [98] reported on rubber addition, which significantly improved deformation and yield strength, as is the case for matrices with higher SiO_2_/Al_2_O_3_ molar ratio and 40 and 50 parts per hundred rubber (phr) rubber. The tensile strength, elongation at break, and hardness of nitrile butadiene rubber (NBR) filled with 40 phr kaolin were satisfactory, and the minimal wear indicated the optimum wear resistance. Additionally, 50 phr kaolin-filled EPDM and CR have adequate elongation at break, hardness, and wear but not tensile strength, which is lower than that of 40 phr kaolin-filled EPDM. However, the durability of rubber is not particularly long. Additionally, rubber cannot handle high temperatures because it may cause a tendency for the material to rupture and degrade, which reduces the composite’s ability to endure tensile strain. In order to create new geopolymer materials from a blend of commercial metakaolins and calcined clays, Selmani et al. [121] focused on valorizing naturally existing clays. Metakaolin MK1 was replaced by metakaolin MK2, which produced different compositions with the following codes: G1, G2 (16%), G2 (33%) and G2 (50%). However, because of impurities, adding natural clay reduced the compressive strength of the geopolymer composites (illite, calcite, iron).

According to Jamil et al. [22], the sluggish rate of the Al content’s dissolution makes it necessary to spend more time to produce kaolin with a strong chemical interaction. A kaolin-GGBS ceramic geopolymer was created by adding ground granulated blast furnace slag (GGBS) to reduce the rate of dissolution. However, research in measuring compressive strength to gauge the brittleness of kaolin geopolymer composites is still limited. In addition, Tiffo et al. [50] reported that, to give the kaolin geopolymer its physical and mechanical properties, researchers substituted amorphous aluminium hydroxide and aluminium oxyhydroxide in varying amounts. As a result, the replacement successfully contributed to the development of heated kaolin-based geopolymers that are thermally stable and have a high compressive strength. The result is not visible, though, until 28 days into the curing process. This is crucial to demonstrate that the geopolymer system has no additional reaction mechanisms because of the removal of kaolin.

Coudert et al.’s [122] study was primarily concerned with the application of an alkali activated fly ash-based binder to improve the engineering properties of soft clay-rich soils and to replace conventional stabilisers (lime or cement). By using optical microscopy, microstructural measurements of the alkali activated fly ash binder treated soil over time were made. In a way like the alkali-activated fly ash binder, after 24 h of curing there are scattered dark patches that look like calcium-rich nodules all over the sample. After 28 days, these nodules are surrounded by larger black zones that are made up of newly formed compounds. At 28 days, hollowed grey nodular structures can be identified as the binder and linked to the breakdown of calcium-rich particles. However, combining micro-indentation with scanning electron imaging would also make it possible to measure regional variations in hardness. Therefore, the degree of calcium particle reactivity can be used to understand how important local microstructural differences are for macroscopic mechanical performances.

In the fresh-state, alkali-activated slurry, Perumal et al. [67] examined how surfactants function as stabilisers for the gas-liquid interface, enhancing the establishment of interconnected porosity utilising impure kaolin. Depending on the paste characteristics, surfactant type, and content, the pore structure produced by direct foaming can have a wide size distribution. Lower strength is generally the result of a less homogenous pore structure. The effect of three different molarities of alkali activator (5 M, 10 M, or 15 M NaOH) and water binder ratio (0.55 and 0.65) on the mechanical property of kaolin-based geopolymer has been described, however the research on the effects of Si/Al ratio and ageing duration has not been covered.

The effects of Micro additions of Fe_2_O_3_ and MgO on the mechanical and physical characteristics of the geopolymer binder were investigated by Kaya et al. [49]. The binder was developed by substituting zeolite for kaolin at percentages of 10%, 20%, and 30%. Additionally, by replacing 4%, 6%, and 8% of the Fe_2_O_3_ and MgO in the binder with zeolite, the quantities of Fe_2_O_3_ and MgO were enhanced. The binder was activated using NaOH that contained 15% Na by weight (Na/binder). Because kaolin has a denser structure due to its lower porosity than zeolite, replacing zeolite with it causes an increase in the unit weight, compressive strength, flexural strength, and UPV of the geopolymer specimens. However, to correspond with the development of hematite (Fe_2_O_3_) and periclase, the formation of sodium aluminosilicate and calcium silicate hydrates as hydration products was not further discussed (MgO). Therefore, these authors were the first to realize the potential of nanoparticles to impart toughness and strength of geopolymer structure. 

### Addition of Zirconia in Ceramic Geopolymers

To increase the strength and toughness of ceramics, for instance, zirconium dioxide (ZrO_2_) may be added. This would be done by taking advantage of the tetragonal to monoclinic phase transformation that is brought on by the presence of a stress field before a break. On the other hand, zirconia brings improvement in compressive strength, fracture toughness, crack deflection, crack bridging, and micro-cracks.

Due to the better chemical and thermal properties to standard additives, nanosized particles are one possibility to increase the mechanical performance of such geopolymers [121,123,124]. Additionally, nanoparticles can function as a filler to lower the nanoporosity at the level of the interfacial transition zone between aggregated particles as well as a catalyst to speed up the geopolymerization reaction [55,124,125]. Table 9 shows the properties of zirconia, including melting point, boiling point, density, and molar mass.

Temperature and time during the sintering process should be studied because they directly affect the grain size, yttrium segregation, and amount of cubic phase in zirconia, which in turn affects its physical, mechanical, and optical properties [14,45,125]. Increasing the sintering temperature increases the grain size of zirconia, which may improve its physical qualities but makes it more susceptible to low-temperature irradiation (LTD). Although Al_2_O_3_ has good hardness, abrasion resistance, and chemical inertness at elevated temperatures, it has relatively low toughness [56,121,123], which leads to early failure. To boost its fracture toughness, Yttria-Stabilized Zirconia (YSZ) is used as a strengthening agent. The product of this mixture is Zirconia-reinforced Alumina (ZTA). It undergoes a phase transformation from tetragonal to monoclinic that results in a transformation strengthening process [45,127].

Greater ZrO_2_ content in a kaolin-based mullite ZrO_2_ composite yielded greater density and flexural strength. Due to the decreased viscosity of the produced glassy phases in the sintered samples [56,128], the presence of ZrO_2_ may have increased the thermal shock resilience of the sample. The proposed method involves adding zirconia to kaolin or metakaolin (calcined kaolinite) in order to produce at high temperatures mullite and zircon (ZrSiO_4_)-based ceramics, according to the following Equation (1) [56]:

3(Al_2_O_3_ · 2SiO_2_) + 4ZrO_2_ → 3Al_2_O_3_ · 2SiO_2_ + 4ZrSiO_4_ (1)

Zircon, which does not suffer any structural change until its dissociation at around 1500 °C, possesses a number of desirable qualities, including a high resistance to alkali corrosion and an extremely low thermal expansion coefficient (4.1 × 10^−6^ °C^−1^) between room temperature and 1400 °C, and a low heat conductivity [56,121,124]. Table 10 shows the impacts of the addition of zirconia in past research.

Phair et al. [129] demonstrated that the incorporation of just 3% mass of zirconia to a geopolymeric matrix significantly increased the compressive strength by 30%. Incorporating 5% or more zirconia, caused considerable brittleness due to the adverse bulk physical effects of extra filler on the 3D polysialate network. However, no clear evidence exists to establish that the absence of zeolite crystallisation is primarily attributable to the high CaO level. Furthermore, Mecif et al. [56] discovered that ZrSiO_4_ production, which occurs at temperatures above 1150 °C, is promoted by the presence of fusing impurities such as K, Fe, Ca, and Mn in clays, as well as a reduction in zirconia particle size. It was also discovered that the rise in the porosity ratio of the final products for zirconia levels more than 20 wt percent was dictated by a decrease in the flux amount due to the reduced clay content. Sintering a mixture of 38 wt% of fine zirconia powder and 62 wt% of the more reactive clay at 1400 °C for 2 h produced ceramics that are mostly composed of zircon and mullite.

Kenawy et al. [130] hypothesized that the comparatively lower density with greater ZrO_2_ contents could be the result of thermal expansion mismatches between ZrO_2_ and the mullite matrix. This may cause interior fissures and a weakened matrix, resulting in a reduced density. Moreover, the higher the ZrO_2_ content, the greater the viscosity of the produced glassy phases and, consequently, the lower the particle diffusion and rearrangement. Regardless, this researcher did not explore the effect on compressive strength. Moreover, the previous researchers theorised that zirconia promotes a 3D polysialate grid structure through the creation of insoluble sodium polysialate, based on research by Zawrah et al. [124] to determine the chemical foundation for the increase in compressive strength. This 3D polysialate minimises the mobility of sodium while maintaining the matrix’s structural integrity and charge balance. To clarify the grain/particle sizes, phases, chemical species, and yttrium distribution, as zirconia materials behave differently at different sintering temperatures, additional research is required.

Geopolymers, which combine some characteristics of organic polymers, cements, and ceramics due to the unusual polycondensed network structure, have attracted a great deal of interest from researchers as a green cementitious material due to the advantageous and distinctive properties. Additional research is necessary to comprehend the properties of kaolin ceramic geopolymers reinforced with zirconia for use in ceramic technology.

## 6. Conclusions and Future Works

Geopolymer material with a low calcium content holds great potential for future ceramic applications because geopolymers have superior characteristics to OPC counterparts and have various performance-related qualities. Even though the characteristics of geopolymer have been thoroughly explored in the literature, there are still certain elements that require additional investigation. Furthermore, it is evident from this brief analysis that addition of zirconia into kaolin geopolymer applications still has a great deal of room for research and improvement. Zirconia-reinforced geopolymer matrices produce materials with enhanced compressive, fracture toughness, crack resistance, and thermal stability, relative to the unreinforced matrix. In conclusion, the essential formulation parameters for geopolymers were assembled and compared based on chemical composition, thermal processing, and consequent mechanical properties. Based on the facts reviewed, it is obvious that additional research must be conducted to optimise formulas for the development of zirconia-reinforced geopolymers with improved characteristics. Several conclusions can be drawn from a review of the existing literature on the properties of geopolymer materials in ceramic industry:

i. Geopolymer materials mentioned above have the potential to be converted into environmentally friendly ceramics because the silico-aluminate used to make them is derived from natural sources and industrial by-products. However, utilizing geopolymer materials derived from kaolin without calcination that have comparable properties to metakaolin-based materials is a significant contribution to sustainability as it reduces energy consumption during the sintering process.
ii. Many factors, such as selection of alkali activator, chemical composition of raw materials and sintering temperature, could have great possibility to produce ceramic geopolymers. Therefore, more data is needed to finally establish a clear relationship between characterization of raw materials and the thermal process.
iii. Typically, geopolymers are weak under tension and fail brittlely. Numerous studies have focused on the inclusion of different types of reinforcement into geopolymers to acquire appropriate mechanical and thermal properties for each application, particularly ceramic applications, to overcome this weakness. The method describing suitable non-destructive testing for evaluating geopolymers alongside the destructive/strength test results is recommended for reviewing in the future.
iv. To impart the strength beyond the standard properties, addition materials are needed. Generally, incorporation of zirconia in kaolin, using a proper alkali activator ratio, chemical composition of raw materials and optimum sintering temperature could increase the compressive strength, and usually result into toughness properties.
v. Although adding inorganic polymer or natural fibres incurs low-cost and is usually flexible and can be used at high content as reinforcement in geopolymers, it is not possible due to its toughness property.
vi. It should be noted that a geopolymer material has demonstrated significant feasibility and application prospects to be used as an environmentally friendly ceramic material, which may be a suitable replacement for the conventional ceramic materials and process in the future.

The significant of this review to the ceramic industry is to produce ceramic geopolymers with high compressive strength at optimal sintering temperature of kaolin. The potential of addition of zirconia may enhance the properties of kaolin geopolymers to reduce energy consumption and increase residual compressive strength without affected the growth of ceramics grains during sintering.

## Figures and Tables

**Table 1:** Mix design of geopolymer materials in ceramic application.

Authors	Raw Materials	Curing Method	Activator Molarity	Formulation
Jamil et al. 2020 [22]	Kaolin (Associated Kaolin Industries Sdn. Bhd., Malaysia)	Curing in 50 × 50 mm mould	NaOH molarity (8 M)	Solid liquid ratio (1, 1.5, 2) Sodium silicate (Na_2_SiO_3_) to sodium hydroxide (NaOH) ratio (4:1) Particle size (kaolin: ~13.3 µm, GGBS: 41.4 µm) Two step sintering temperature (1st: 500 °C, 2nd: 900 °C)
Ma et al. 2021 [46]	Kaolin (95%, Fengxian Reagent Factory, China) SCWS (99.9%, Beijing, Forsman Technology Co., Ltd., China, d = 500 nm, l = 13 µm) Silica sol (40%, Jiangsu Xiagang, Indus, China)	Cast into polystyrene containers and cured at 60 °C the incubator (7 day)	H_2_O/K_2_O = 11 (mole ratio)	Calcining kaolin at 800 °C Stirring 24 h on the rotating ball mill at 60 °C Treated in a tube furnace (RHTH120/600/18, Nabertherm, Germany) at (1100–1200 °C) SiO_2_/Al_2_O_3_ = 4, SiO_2_/K_2_O = 4 SCWS contents (0.5 wt%, 1 wt%, 2 wt%, 3 wt%, and 4 wt%)
Yun Ming et al. 2017 [48]	Metakaolin	Pre curing (80 °C, 4 h) Curing (RT, 40, 60, 80, 100 °C at 6, 12, 24, 48, 72 h) Curing day (7, 28 day)	NaOH molarity (8 M)	Kaolin (sintered at 800 °C for 2 h) Na_2_SiO_3_ to NaOH ratio (0.8 and 0.2)

**Table 2:** Kaolin geopolymer on various ceramic applications.

Authors	Raw Materials	Curing Process	Application
Kovarik et al. 2017 [9]	Kaolin	Expose to 1000 °C for 30 min, then let it cool at room temperature.	Ceramic grog
Keppert et al. 2020 [60]	Red kaolin	Thermal curing at 60 °C	Cementitious materials
Mohamad Zaimi et al. 2020 [61]	Kaolin	24 h curing at a temperature of 80 °C	Electronic packaging industries
Kovarik et al. 2021 [62]	Calcined kaolin and blast furnace slag	Sintering temperature 1300 °C for 3 h. Curing temperature 70 °C	Ceramic foam
Cheng et al. 2021 [15]	Coal-series kaolin	At a rate of 5 °C/min, the maximum temperature was kept at 600 °C, 650 °C, and 700 °C for 2 h. The crucibles were taken out of the furnace and the calcined kaolin was quickly cooled to room temperature.	Geopolymeric cement materials
Aziz et al. 2021 [63]	Natural perlite and kaolinic clay	Placed until the test age in a curing chamber with a relative humidity of > 90%	Ceramic insulator
Sarde et al. 2022 [64]	Kaolin	Calcined at 600 °C for 2 h	Electoceramic (Dielectric character)
Marsh et al. 2019 [65]	Kaolin	Pre-dried and allowed to cool in a 105 °C oven.	Soil construction
Wang et al. 2022 [66]	Nano-ZnO/melamine polyphosphate (MPP) and silica fume clay	Thermal acceleration rate of 10 °C⋅min^−1^ from 40 to 1000 °C under a pure N_2_ atmosphere.	Ceramic coating

**Table 3:** Si and Al content of kaolin for geopolymer synthesis.

Authors	Content (wt%)		Particle Size, D_50_ (µm)	Surface Area, (m^2^/g)
	SiO_2_	Al_2_O_3_		
Kovarik et al. 2017 [9]	52.1	41.9	4.0	13.0
Borges et al. 2017 [75]	54.5	44.2	4.5	N/A
Belmokhtar et al. 2017 [76]	53.6	42.2	4.8	6.2
Lahoti et al. 2017 [77]	53.0	43.8	1.3	N/A
Belmokhtar et al. 2018 [51]	47.2	37.12	6.20	4.72
Kwasny et al. 2018 [78]	32.04	24.99	N/A	1.57
Marsh et al. 2019 [2]	57.76	22.85	2.0	17.6
	60.73	24.05	2.0	33.7
	60.20	11.60	2.0	36.9
Jamil et al. 2020 [22]	54.0	31.7	13.3	N/A
Matalkah et al. 2020 [79]	52.1	26.2	Less than 100	2.67
Nnaemeka et al. 2020 [80]	45.3	38.38	N/A	N/A
Tiffo et al. 2020 [50]	38.00	40.10	90	N/A
Rania and Samir 2021 [81]	48.21	39.85	1–80	4.78
Aziz et al. 2021 [63]	55.14	28.52	63	N/A
Mehmet et al. 2022 [49]	70.32	18.87	N/A	N/A
Alexandre and Lima 2022 [26]	36.3	34.9	2	N/A
	47.08	39.19	2	

**Table 4:** Previous research on kaolin and alkali activator.

Authors	Activator		Molarity	Raw Material
Prasanphan et al. [86]	NaOH	Na_2_SiO_3_	10 M NaOH	Kaolin
Jamil et al. 2020 [22]	NaOH	Na_2_SiO_3_	6 to 8 M NaOH	
Aziz et al. [63]	NaOH	Na_2_SiO_3_	8 M NaOH	
Alexandre et al. 2022 [26]	KOH		16 M of KOH	

**Table 5:** Sintering temperature from past research.

Authors	Raw Material	Sintering Temperature Range (°C)	Optimum Sintering Temperature Range (°C)	Phase Formation
Naghsh and Shams 2017 [89]	Kaolin	400–800	600	Major crystalline phase is kaolinite. Amorphous phase in which the kaolin (peak at 20–40°)
Sornlar et al. 2021 [90]		600	600	Amorphous phase increase with certain crystalline phases still present (illite and quartz)
Alexandre and Lima 2022 [26]		750	750	Neoformation of hematite, from the dehydroxylation of goethite
Majdoubi et al. 2021 [91]		300–1100	800	Undergo a significant transformation from amorphous to entirely crystalline
Merabtene et al. 2019 [92]		800	800	Muscovite is transformed into Anorthite through its reaction with CaO, which also results in the development of other minerals like Quartz and Leucite.
Villaquirán and Mejia 2018 [93]		300–1500	900	Below 900 °C—no obvious structure change Exceed 900 °C—formation of macroporous mullite ceramic
Jamil et al. 2020 [22]		200–1200	900	Kaolinite phase at 2θ of ~13°, ~25°, and ~26° Disappearance of the gehlinite phase Phases of akermanite and albite in SL ratio 2
Liew et al. 2017 [48]		900–1300	1200	Major crystalline phase is kaolinite. Formation of crystalline nepheline

**Table 6:** Compressive strength and factors affecting.

Authors	Raw Material	Compressive Strength	Significant Design Parameter	Factors Affecting Compressive Stress
Hajkova, 2018 [19]	Calcined kaolinite claystone	68 MPa at 28 days curing	Constant weight ratio water glass: kaolinite: calcium hydroxide Water glass density (1.2, 1.3, 1.4, 1.5, and 1.6 g·cm^−3^)	Lower total pore volume and increasing the compressive strength (at higher density of water glass-1.5 g·cm^−3^)
Matalkah et al., 2020 [79]	Kaolin	48 MPa	Added calcium oxide and sodium hydroxide (0, 5, 10, 15, and 20% by weight of kaolin) 5 wt.% sodium hydroxide and 10 wt.% calcium oxide	Ca makes C-S-H phases more likely, which could make the geopolymer paste denser. NaOH could make it easier for Si and Al to leach out of the kaolin particles and into the solutions, which led to more geopolymerization. Formation of N-A-S-H gel
Tiffo et al., 2020 [50]	Kaolin	29.1 MPa heated at 1100 °C	Used aluminium hydroxide and oxyhydroxide to replace kaolin (30% by mass)	Formation of stable crystalline phases Nepheline and carnegieite are partially dissolved, which lead to closed pores and a drop in compressive strength.
Ababneh et al., 2020 [106]	Kaolin	7-day 19.83 MPa (heat cure) 16.47 MPa (room-cure)	62.5 wt.% kaolin, 30 wt.% calcium oxide, 5 wt.% sodium carbonate and 2.5 wt.% sodium silicate	Due to the loss of water, a high curing temperature could also make the resulting geopolymer matrix more porous. Presence of calcium oxide in the aluminosilicate generate C-S-H gel

**Table 7:** Chemical composition of kaolin geopolymer.

Authors	Al_2_O_3_	SiO_2_	MgO	Fe_2_O_3_	SO_3_	K_2_O	Na_2_O	CaO	TiO_2_	P_2_O_5_	LOI
Heah et al. 2012 [109]	31.7	54.0	0.11	4.89	0	6.05	0	0	1.41	0	1.74
Hajkova 2018 [19]	41.45	52.03	0.13	1.05	0.20	0.79	0	0.15	1.62	0.06	2.52
Belmokhtar et al. 2018 [51]	37.12	47.2	0.39	0.83	0	2.2	0.05	0.03	0.04	0	0
Mehmet et al. 2022 [49]	18.87	70.32	0.32	0.58	1.33	0.87	0.04	1.44	-	0.1	6.03

**Table 8:** Addition as a reinforcement in ceramic composite.

Authors	Raw Materials	Addition	Percentage/Ratio Addition	Finding Descriptions
Wu and Tian, 2013 [98]	Kaolin	Rubber composites(NR, SBR, BR, NBR, EPDM, MVQ, and CR)	40 parts per hundred rubber (phr) and 50 parts per hundred rubbers (phr)	Superior tensile strength and weaker elongation at break Plate-like structure of kaolin helps rubber release heat and makes rubber composites more stable at high temperatures.
Selmani et al., 2017 [121]	Kaolin	Commercial Metakaolin	0%, 16%, 33% and 50%	Increasing the metakaolin percentage confirms the existence of different networks. More reinforcements (illite and mica) caused more networks to form and more impurities (illite and calcite) to be coated by the excess alkaline solution.
Jamil et al., 2020 [22]	Kaolin	Ground granulated blast furnace slag (GGBS)	Kaolin:GGBS (4:1 wt.%)	Accelerate the geopolymer’s setting time and contributed to the phase transformation of sintered kaolin-GGBS geopolymer.
Tiffo et al. 2020 [50]	Kaolin	Amorphous aluminium hydroxide and aluminium oxyhydroxide	0%, 10%, 20% and 30% by mass	Enhances compressive strength and thermal stability 30% by mass of aluminium oxyhydroxide heated at 1100 °C, compressive strength of 29.1 MPa 10% by mass of amorphous aluminium hydroxide gave 60.2 MPa at 1150 °C. Formation of stable crystalline phases
Coudert et al., 2021 [122]	Kaolin	Fly ash	10%, 20% and 40% of fly ash with reference to dry mass of solids (fly ash + kaolin).	Porosity is less because small kaolinite platelets fill the pores. Decrease in the soil’s ability to be compressed and an increase in the yield stress
Perumal et al., 2021 [67]	Kaolin	Surfactants (Hydrogen peroxide, chemical that lowers surface tension)	5, 10, 15 M NaOH Water binder ratio (0.55 and 0.65)	Strength improvement mainly by bubble stabilization avoiding the bubble coalescence and, by reducing the pore size Surfactants and H_2_O_2_ bringing down the viscosity values by 20–60% and the effect is higher at higher H_2_O_2_ dosage
Kaya et al., 2022 [49]	Kaolin	Zeolite	Replacing 10%, 20%, and 30% kaolin with zeolite	4%, 5%, and 6% increase in unit weight Increased compressive strength and flexural strength 3%, 7%, and 12% increase in ultrasonic pulse velocity (UPV) of the geopolymer specimens due to the formation of dense structure owing to lower porosity of kaolin than zeolite

**Table 9:** Zirconia properties [126].

Properties	Value
Melting Temperature (°C)	2715 4300 5.68 123.2
Boiling temperature (°C)	
Density (g/m^2^)	
Molar mass (g/mol)	

**Table 10:** Impact of the addition of zirconia in past research.

Authors	Percentage Addition (%)	Raw Materials	Properties Improvement and Mechanism Reaction
Phair et al., 2000 [129]	0, 1%, 3%, 5%, 7% by mass of FA	Fly ash	Increase compressive strength Chemically, non-aluminosilicate materials are thought to be based on zirconia’s ability to make insoluble sodium polysialate, which then forms a 3D polysialate grid structure. Zeolite production could not occur using zirconia as a nucleation germ or template.
Mecif et al., 2010 [56]	0%, 10%, 20%, 30% and 40% wt	Metakaolin	High thermal stability, low thermal expansion, and conductivity High creep resistance associated with strong strength, and fracture toughness The amount of flux in the mixture cannot get any denser because the clay content is decreased, and silica is being used up when ZrSiO_4_ is made. Disappearance of cristobalite occurs during zircon formation
Kenawy et al., 2016 [130]	0, 5, 10, 15 and 20 wt%	Calcined kaolin at 1000 °C for 2 h	High thermal shock resistance and flexural strength Reduction in the viscosity of the glassy phases that develop in sintered samples Glassy phase, which might help serious faults repair or make materials appear more durable throughout the sintering process Continuous solid solution at the grain boundary between ZrO_2_ and mullite strengthens the grain-boundary mechanism
Zawrah et al., 2018 [124]	0, 10%, 15% by weight of metakaolin	Metakaolin	Compressive strength was increased (10% gives 74 MPa) No new phases were produced because zircon did not take part in the geopolymerization process. Instead, it filled the spaces between the polysialate networks.

## Data Availability

Not applicable.
